# Sociodemographic And Health Characterization Of The South Texas Alzheimer’s Disease Research Center Cohort

**DOI:** 10.1093/geroni/igaf122.4201

**Published:** 2025-12-31

**Authors:** Silvia Mejia-Arango, Jeremy A Tanner, Campbell Sullivan, Ashley LaRoche, Jennifer Del Bosque, Rosa Pirela, Gladys Maestre, Sudha Seshadri

**Affiliations:** University of Texas Rio Grande Valley, Harlingen, Texas, United States; University of Texas Health Sciences Center at San Antonio, San Antonio, Texas, United States; University of Texas Health Sciences Center at San Antonio, San Antonio, Texas, United States; University of Texas Health Sciences Center at San Antonio, San Antonio, Texas, United States; UT Health San Antonio, San Antonio, Texas, United States; University of Texas Rio Grande Valley, Harlingen, Texas, United States; University of Texas Rio Grande Valley, Edinburg, Texas, United States; University of Texas Health Sciences Center at San Antonio, San Antonio, Texas, United States

## Abstract

The South Texas ADRC (STAC) is the only Texas-based ADRC with three sites—San Antonio, Laredo, and the Rio Grande Valley—and leads in Hispanic participant data (about 60%) among the 36 active ADRCs. To better understand the characteristics of our cohort, we analyzed differences in sociodemographic and health conditions by ethnicity, as well as their role as predictors of cognitive status. A total of 773 participants enrolled in STAC who completed a standardized clinical evaluation protocol used in the ADRCs, were included in the analysis. Research participants were aged 71 ± 9.6 years; 60% were female; the mean years of education were 14.6 ± 3.6 years; and 59% were Hispanic. Rates of dementia were significantly higher among non-Hispanics (30% vs. 17.4%), while MCI rates were not significantly different (24.9% vs. 28.3%). Additionally, there were no differences in cognitive impairment not classified as MCI (1% vs. 3.5%). We found a significant trend towards worse conditions among Hispanic participants compared with non-Hispanics. Hispanics had significantly fewer years of education; higher socioeconomic disadvantage at the state level based on the adversity index (ADI), and higher rates of diabetes (32%), hypertension (72.9%), and hypercholesterolemia (64.3%). Predictors of dementia among Hispanics were older age, lower years of education, and being male. Among non-Hispanics, older age and being male were the only significant predictors of dementia. Different recruitment and enrollment strategies across the three STAC sites, leading to high heterogeneity among subsamples, are discussed as possible drivers of our results.

